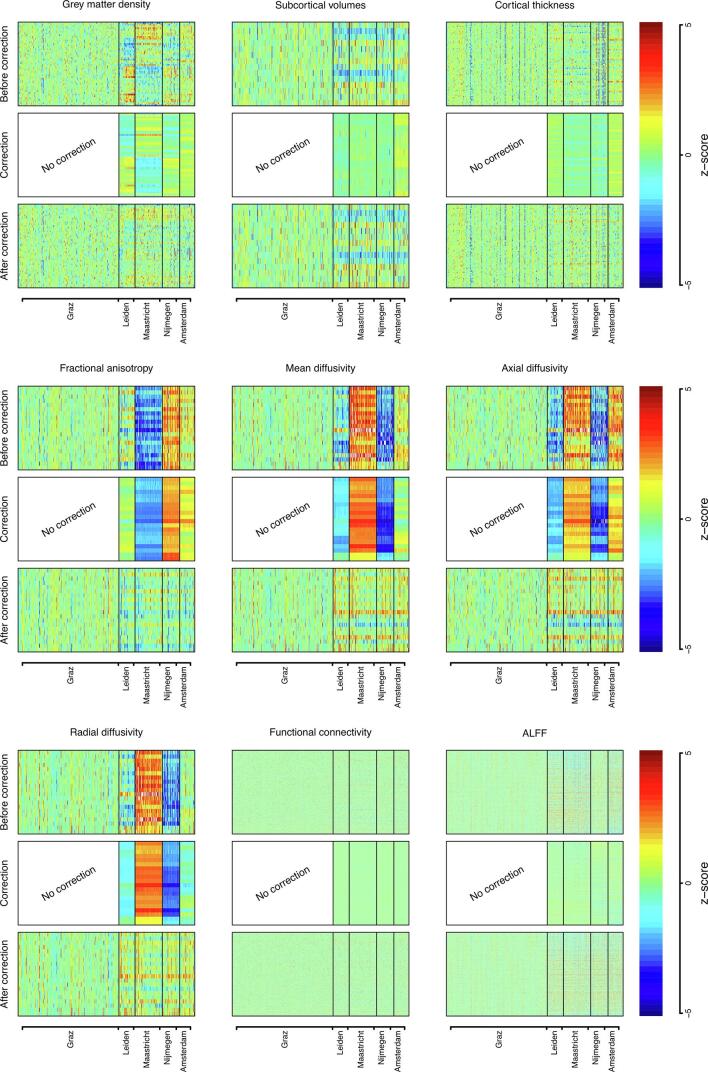# Pre-trained MRI-based Alzheimer's disease classification models to classify memory clinic patients

**DOI:** 10.1016/j.nicl.2020.102303

**Published:** 2020-06-04

**Authors:** Frank de Vos, Tijn M. Schouten, Marisa Koini, Mark J.R.J. Bouts, Rogier A. Feis, Anita Lechner, Reinhold Schmidt, Mark A. van Buchem, Frans R.J. Verhey, Marcel G.M. Olde Rikkert, Philip Scheltens, Mark de Rooij, Jeroen van der Grond, Serge A.R.B. Rombouts

**Affiliations:** a Institute of Psychology, Leiden University, the Netherlands; b Department of Radiology, Leiden University Medical Center, the Netherlands; c Leiden Institute for Brain and Cognition, the Netherlands; d Department of Neurology, Medical University of Graz, Austria; e Department of Psychiatry and Neuropsychology, School for Mental Health and Neuroscience (MHeNS), Alzheimer Centrum Limburg, Maastricht University, the Netherlands; f Department of Geriatric Medicine, Radboudumc Alzheimer Centre, Radboud University Medical Center, Nijmegen, the Netherlands; g Department of Geriatric Medicine, Radboudumc Alzheimer Centre, Donders Institute for Medical Neurosciences, Radboud University Medical Center, Nijmegen, the Netherlands; h Department of Neurology, Alzheimer Center Amsterdam, Amsterdam Neuroscience, Amsterdam UMC, Vrije Universiteit Amsterdam, Amsterdam, the Netherlands

**Keywords:** Alzheimer’s disease, Mild cognitive impairment, Subjective memory complainers, Anatomical MRI, Diffusion MRI, Resting state fMRI, Classification

## Abstract

Anatomical magnetic resonance imaging (MRI), diffusion MRI and resting state functional MRI (rs-fMRI) have been used for Alzheimer’s disease (AD) classification. These scans are typically used to build models for discriminating AD patients from control subjects, but it is not clear if these models can also discriminate AD in diverse clinical populations as found in memory clinics.

To study this, we trained MRI-based AD classification models on a single centre data set consisting of AD patients (N = 76) and controls (N = 173), and used these models to assign AD scores to subjective memory complainers (N = 67), mild cognitive impairment (MCI) patients (N = 61), and AD patients (N = 61) from a multi-centre memory clinic data set. The anatomical MRI scans were used to calculate grey matter density, subcortical volumes and cortical thickness, the diffusion MRI scans were used to calculate fractional anisotropy, mean, axial and radial diffusivity, and the rs-fMRI scans were used to calculate functional connectivity between resting state networks and amplitude of low frequency fluctuations. Within the multi-centre memory clinic data set we removed scan site differences prior to applying the models.

For all models, on average, the AD patients were assigned the highest AD scores, followed by MCI patients, and later followed by SMC subjects. The anatomical MRI models performed best, and the best performing anatomical MRI measure was grey matter density, separating SMC subjects from MCI patients with an AUC of 0.69, MCI patients from AD patients with an AUC of 0.70, and SMC patients from AD patients with an AUC of 0.86. The diffusion MRI models did not generalise well to the memory clinic data, possibly because of large scan site differences. The functional connectivity model separated SMC subjects and MCI patients relatively good (AUC = 0.66). The multimodal MRI model did not improve upon the anatomical MRI model.

In conclusion, we showed that the grey matter density model generalises best to memory clinic subjects. When also considering the fact that grey matter density generally performs well in AD classification studies, this feature is probably the best MRI-based feature for AD diagnosis in clinical practice.

## Highlights

• Multimodal MRI AD classification models were pre-trained on AD patients and controls.
• Generalisation of these models was tested on a multi-centre memory clinic data set.
• AD scores were assigned to AD patients, MCI patients and memory complainers.
• Anatomical MRI performed better than diffusion MRI and resting state fMRI.
• Combining imaging modalities did not improve the results over anatomical MRI only.

## 1 Introduction

Early diagnosis of Alzheimer’s disease (AD) is important, because it enables patients and caregivers to prepare for disease progression (Prince et al., 2011). It is also beneficial for drug research, because early phase AD patients are more likely to be susceptible to medication (Cummings et al., 2016). Whereas the diagnosis of progressed AD is feasible (Frisoni et al., 2010), early identification of AD is still problematic (Frisoni et al., 2017).

Amyloid and tau pathology are hypothesised to occur early in AD (Jack et al., 2010) and tau-PET and amyloid-PET are hypothesised earliest AD biomarkers (Blennow and Zetterberg, 2018). However, for clinical studies Magnetic resonance imaging (MRI) scans are advantageous, because they are often available, they are non-invasive and they are relatively cheap. Further, functional MRI measures have been hypothesised to change in early AD as well (Buckner et al., 2005, Sperling, 2011).

Magnetic resonance imaging (MRI) has been used to characterise brain changes that occur in AD. Most prominently, AD is characterised by grey matter atrophy, starting in the hippocampus (Morra et al., 2010), and later extending to other brain regions, including subcortical structures and the medial temporal lobe (Jack et al., 2004, Seeley et al., 2009). The location and extent of grey matter atrophy can be determined using anatomical MRI. Brain alterations in AD patients also involves white matter integrity (Douaud et al., 2011), which can be shown by diffusion MRI. In addition, AD patients show altered functional connectivity between brain regions (Agosta et al., 2012, Binnewijzend et al., 2012), measured using resting state functional MRI (rs-fMRI).

However, these group differences are not necessarily useful in a clinical setting, since many AD markers have also been observed in healthy ageing (Salat et al., 1999). AD markers are only helpful in a clinical setting if they can accurately discriminate AD patients from non-affected subjects at the individual level. The focus of research on MRI biomarkers for AD has therefore shifted from the detection of group differences toward disease classification. MRI-based classification studies have progressed by using machine learning techniques, in which many predictors can be combined into one predictive model. This has led to good AD classification results for structural MRI (Cuingnet et al., 2011, Davatzikos et al., 2011, de Vos et al., 2016), diffusion MRI (Dyrba et al., 2013, Schouten et al., 2017) and resting state fMRI (Challis et al., 2015, Chen et al., 2011, de Vos et al., 2018). Moreover, combining these three MRI modalities can further improve the classification accuracy (Schouten et al., 2016).

Although these results are promising, MRI-based classification models still have to surmount at least two problems. First, most MRI-based AD classification studies have used scans of AD patients and healthy elderly controls, and other studies have used scans of mild cognitive impairment (MCI) patients to predict AD conversion (see for an overview Rathore et al., 2017). These models are trained specifically for these classification problems, but it is not clear whether these models can also discriminate AD in diverse clinical populations as found in memory clinics. It is thus important to evaluate the generalisability of MRI-based AD classification models to diverse clinical populations. Second, MRI scans are susceptible to scanner effects (Ewers et al., 2006, Takao et al., 2014, Zhu et al., 2011). This is problematic when a classification model is trained with MRI scans from one scanner, and applied to MRI scans from another scanner. To be clinically useful, AD classification models should be robust to scanner effects.

We will study to which extent MRI-based AD classification models generalise to a diverse patient population. This study is novel on 2 important points. Firstly, we will apply an AD classification model to a group of memory clinic patients, who are prone to AD. This is more clinically relevant than classifying AD from healthy controls, but also much more challenging. Second, we will use both structural MRI, diffusion MRI and rs-fMRI scans. This enables a comparison between these imaging modalities, and the use of a multimodal MRI classification model. We will use two different data sets. The first data set consists of AD patients and healthy controls, and will be used for training MRI-based AD classification models. These classification models will then be applied to the second data set, that consists of a diverse patient population collected in four different memory clinics. The memory clinic data set contains AD patients, MCI patients and subjects with subjective memory complaints (SMC). We expect that AD patients will have a higher likelihood of being classified as AD patient than both other groups. Furthermore, we expect this to be higher for MCI patients than for SMC subjects, because MCI is often an early stage of AD.

## 2 Methods

### 2.1 Participants

#### 2.1.1 Training data

The training data were collected at the medical university of Graz in Austria, and consisted of 76 clinically diagnosed probable AD patients and 173 cognitively normal elderly controls (see Table 1). The AD patients were part of the prospective registry on dementia (PRODEM; see also Seiler et al., 2012). The inclusion criteria for PRODEM are: dementia diagnosis according to DSM-IV criteria (American Psychiatric Association, 2000), AD diagnosis according to the NINCDS-ADRDA Criteria (McKhann et al., 2011), non-institutionalisation or need for 24-h care, and the availability of a caregiver who agrees to provide information on the patients’ and his or her own condition. Patients were excluded if co-morbidities were likely to preclude successful completion of the study. Informed consent was obtained from all patients and their caregivers. We only included patients for which anatomical MRI, diffusion MRI and rs-fMRI were available. The controls were scanned at the same scanning site, over the same period, with the same scanning protocol as the AD patients as a part of the Austrian stroke prevention study. The Austrian Stroke Prevention Study is a community-based cohort study on the effects of vascular risk factors on brain structure and function in elderly participants without a history or signs of stroke and dementia on the inhabitants of Graz, Austria (Schmidt et al., 1994, Freudenberger et al., 2016). Informed consent was obtained from all participants.

**Table 1:** Sample demographics.

	Training data		Memory clinic data		
	Controls	AD patients	SMC	MCI	AD patients
N	173	76	67	61	61
Sex (♂/♀)	74/99	30/46	48/19	35/26	34/27
Age	66.1 ± 8.7	68.6 ± 8.6	63.2 ± 10.3	69.7 ± 8.3	72.5 ± 9.2
Years of education	11.5 ± 2.8	10.8 ± 3.2	11.2 ± 3.4	11.2 ± 3.4	10.6 ± 3.5
MMSE	27.5 ± 1.8	20.4 ± 4.5	28.2 ± 1.6	26.9 ± 2.3	24.0 ± 2.7
CDR	–	0.82 ± 0.34	0.34 ± 0.25	0.53 ± 0.15	0.78 ± 0.25
GDS	2.0 ± 2.4	2.7 ± 2.6	3.7 ± 2.8	3.0 ± 2.4	3.2 ± 2.8

Descriptives are presented as frequencies for the categorical variables and as mean ± standard deviation for the other variables. AD = Alzheimer’s disease, SMC = Subjective memory complainers, MCI = Mild cognitive impairment, MMSE = mini mental state examination, CDR = clinical dementia rating, GDS = geriatric depression scale.

#### 2.1.2 Memory clinic data

The memory clinic data (see Table 1) are part of the Leiden-Alzheimer research Nederland (LeARN) project (Handels et al., 2012, Jansen et al., 2017), and consisted of 61 possible or probable AD patients, 61 MCI patients and 67 SMC subjects. The AD diagnosis was according to the NINCDS-ADRDA Criteria (McKhann et al., 2011), and the MCI diagnosis was according to the core clinical criteria for MCI due to AD (Albert et al., 2011). Subjects that did not meet the criteria for either AD or MCI were included in the SMC group. LeARN is a multi-center collaboration of four memory clinics in the Netherlands; Leiden, Maastricht, Nijmegen and Amsterdam (see suppl. Table 1 for the demographics stratified over center). The inclusion criteria for LeARN are: subjective and/or objective memory complaints, suspicion of having a primary neurodegenerative disease, a Mini-Mental State Examination ≥20, clinical dementia rating between 0 and 1 and the availability of a reliable informer or proxy who visits or contacts the patient at least once a week. We only included patients for which anatomical MRI, diffusion MRI and rs-fMRI were available and excluded patients diagnosed with MCI not due to AD or dementia not due to AD (e.g. vascular dementia or frontotemporal dementia). Informed consent was obtained from both the patient and the informal caregiver.

### 2.2 MR acquisition

The subjects in the training data were scanned on a Siemens TrioTim 3T scanner at the Graz medical center. The memory clinic subjects were scanned on a Philips Achieva 3T scanner at the Leiden University Medical Center, a Philips Achieva 3T scanner at the Maastricht University Medical Center, a Siemens TrioTim 3T scanner at the Nijmegen University Medical Center and a GE Signa HDxt 3T scanner at the VU university medical center in Amsterdam. The MRI sequence parameter settings are listed in Table 2.

**Table 2:** MRI sequence parameter settings per scan site.

	Slices	TR (ms)	TE (ms)	Flip angle (°)	Matrix size (voxels)	Voxel size (mm)		
**anatomical MRI**								
Graz	176	1900	2.2	9	256 × 256	1.00 × 1.00 × 1.00		
Leiden	180	9.8	4.6	8	288 × 288	0.78 × 0.78 × 1.00		
Maastricht	180	8.2	3.7	8	240 × 240	1.00 × 1.00 × 1.00		
Nijmegen	192	2300	4.7	12	256 × 256	1.00 × 1.00 × 1.00		
Amsterdam	176	7.8	3.0	12	256 × 256	0.94 × 0.94 × 1.00		
**diffusion MRI**							**Directions**^a^	**b0 scans**
Graz	50	6700	95	90	125 × 125	2.00 × 2.00 × 2.50	12^b^	4
Leiden	70	8250	80	90	128 × 128	2.00 × 2.00 × 2.00	61	1
Maastricht	70	8250	80	90	128 × 128	2.00 × 2.00 × 2.00	61	1
Nijmegen	81	13,000	102	90	128 × 128	2.00 × 2.00 × 2.00	30	1
Amsterdam	45	13,000	94	90	128 × 128	2.00 × 2.00 × 2.40	30	1
**rs-fMRI**							**Volumes**	
Graz	40	3000	30	90	64 × 64	3.00 × 3.00 × 3.00	150	
Leiden	38	2200	30	80	80 × 80	2.75 × 2.75 × 3.00	200	
Maastricht	38	2200	30	80	112 × 112	2.00 × 2.00 × 2.50	200	
Nijmegen	49	2380	30	90	64 × 64	3.50 × 3.50 × 3.50	110	
Amsterdam	34	1800	35	80	64 × 64	3.30 × 3.20 × 3.00	202	

a All diffusion directions were acquired with a b value of 1000.

b The diffusion directions were acquired four times.

### 2.3 MRI preprocessing

The MRI data of all subjects were preprocessed using the FMRIB Software Library (FSL version 5.0; Jenkinson et al., 2012, Smith et al., 2004). For the anatomical MRI scans we applied brain extraction and bias field correction. For the diffusion MRI scans, we applied brain extraction and eddy current correction. For the rs-fMRI data, this included brain extraction, motion correction, a temporal high pass filter with a cutoff point of 100 s, 3 mm FWHM spatial smoothing, and non-linear registration to standard MNI152 space. Additionally, we used ICA-AROMA to automatically identify and remove noise components from the fMRI time course (Pruim et al., 2015). ICA-AROMA adequately removes motion related noise from fMRI data, without the need for removing volumes with excessive motion (Parkes et al., 2018).

### 2.4 Anatomical MRI features

We used both the FSL and Freesurfer software packages to analyse the anatomical MRI scans, because they have different approaches to calculate measures of grey matter atrophy. These approaches are complementary to each other, and combining them improves the accuracy of AD classification (de Vos et al., 2016).

#### 2.4.1 Grey matter density

We used voxel based morphometry (VBM; Ashburner and Friston, 2000) in FSL (Jenkinson et al., 2012, Smith et al., 2004) to calculate grey matter density. This includes segmentation of the brain-extracted images into grey matter, white matter, and cerebral spinal fluid (CSF), and non-linear registration of the grey matter images to the ICBM-152 grey matter template. We then calculated weighted averages of the voxel-wise grey matter density values within the 48 regions of the probabilistic Harvard-Oxford cortical atlas, yielding 48 grey matter density values per subject.

#### 2.4.2 Subcortical volumes

We used the FMRIB’s Integrated Registration and Segmentation Tool (FIRST; Patenaude et al., 2011) to calculate the volumes of the subcortical structures and we corrected the volumes for intracranial volume. This yielded 14 subcortical volume features per subject (thalamus, caudate, putamen, pallidum, hippocampus, amygdala, and accumbens for both hemispheres).

#### 2.4.3 Cortical thickness

We used the Freesurfer software package (Dale et al., 1999, Fischl et al., 1999) to calculate cortical thickness. This includes intensity normalisation of the brain-extracted image to obtain an image with high contrast to noise ratio. This image is used to locate the boundaries between grey matter, white matter and CSF. Subsequently, a triangular mesh is constructed around the white matter surface, and this mesh is deformed outwards to create a grey matter surface that closely follows the boundary between grey matter and CSF. Cortical thickness is defined as the distance between the white matter surface and the grey matter surface. The image is registered to the Freesurfer common template using the image’s cortical folding pattern, and the neocortex is parcellated into the 68 neocortical regions (34 regions for each hemisphere) of the Desikan-Killiany atlas (Desikan et al., 2006). This yielded 68 cortical thickness features per subject.

### 2.5 Diffusion MRI features

We used the diffusion MRI scans to calculate fractional anisotropy (FA), mean diffusivity (MD), axial diffusivity (DA), and radial diffusivity (DR). First, we used DTIFIT in FSL (Jenkinson et al., 2012, Smith et al., 2004) to fit a diffusion tensor model at each voxel to calculate voxel-wise FA, MD, DA and DR images for each subject. Then we projected subjects’ FA, MD, DA and DR images onto the FMRIB58_FA mean FA image using tract based spatial statistics (TBSS; Smith et al., 2006). Finally, we calculated weighted averages of the FA, MD, DA and DR values within the 20 regions of the probabilistic JHU white-matter tractography atlas, yielding 20 features for FA as well as MD, DA and DR.

### 2.6 rs-fMRI features

#### 2.6.1 Functional connectivity

Functional connectivity was calculated between resting state networks (RSNs) as obtained by an independent component analysis (ICA). First, we used only the training sample to obtain 70 RSNs using temporal concatenation ICA in FSL MELODIC (Beckmann and Smith, 2004). Then, for all subjects we registered the ICA component weight maps to subject space, weighted them by the subject specific grey matter density maps, and multiplied them with the functional data. Subsequently, we calculated the mean time courses for the 70 components and used these for the FC analysis. We calculated sparse partial correlations using the Graphical Lasso algorithm (Friedman et al., 2008), with ƛ=100 (Smith et al., 2011). For each participant we thus calculated a 70 by 70 sparse partial correlation matrix yielding (70 * 69)/2 = 2415 features.

#### 2.6.2 Amplitude of low frequency fluctuations

To calculate the amplitude of low frequency fluctuations (ALFF; Biswal et al., 2010, Zang et al., 2007), we used the REST software package (Song et al., 2011). ALFF was defined as the power within the 0–0.1 Hz frequency band. For standardisation purposes we divided the voxels’ ALFF values by the mean ALFF within a subjects’ whole brain (Zang et al., 2007). The whole brain voxel-wise ALFF maps consist of 139,712 values.

### 2.7 Correction for age

We regressed out the age effects from the features. To this end we first used the healthy controls from the training sample to estimate ‘normal’ age effects for all features. Then we used these estimated age effects to regress out the age effects for all subjects.

### 2.8 Correction for scan site within the memory clinic data

We corrected for scan site effects within the memory clinic data using ComBat (Johnson et al., 2007). ComBat is validated for structural MRI data (Fortin et al., 2018), diffusion MRI data (Fortin et al., 2017), and rs-fMRI data (Yu et al., 2018). ComBat fits a linear model of location and scale for each feature, making the assumption that sites have both an additive and multiplicative effect on the data. It uses empirical Bayes to improve the estimation of the model parameters. The model furthermore makes the assumption that the expected value of a feature can be modelled by both the site effect, and biological and demographical factors. ComBat thus removes the unwanted site effects, while it preserves the variation that is associated with the biological and demographical factors. We included age, sex, years of education, clinical label, and MMSE score as factors in the ComBat model.

We did not correct for scan site differences between the training data and the memory clinic data, because the training data consists of different clinical labels (healthy controls and probable AD) than the memory clinic data (SMC, MCI and possible/probable AD). It is therefore not possible to decide whether differences between these data sets should be attributed to scan site differences, or to differences in clinical groups.

### 2.9 Statistical analysis

The nine different MRI feature groups, along with the number of features per group are listed in Table 3. These feature groups were used separately in nine different AD classification models, and combined into an anatomical MRI, diffusion MRI, rs-fMRI and multimodal AD classification model. All features were normalised prior to the statistical analyses.

**Table 3:** MRI features.

	# of features
**Anatomical MRI features**	
Grey matter density	48
Subcortical volumes	14
Cortical thickness	68
**Diffusion MRI features**	
Fractional anisotropy	20
Mean diffusivity	20
Axial diffusivity	20
Radial diffusivity	20
**resting state fMRI features**	
Functional connectivity	2,415
Amplitude of low frequency fluctuations	139,712

#### 2.9.1 Penalised logistic regression within the training data

The training data was used to fit AD classification models. We used logistic regression to predict the true class of the subjects. In logistic regression, the outcome variable is dichotomous (0 for healthy controls and 1 for AD patients), and the predicted scores are continuous between 0 and 1. The subjects’ predicted scores are adopted as AD scores. To prevent overfitting, we used penalised logistic regression techniques that put penalties on the regression weights, such that only the most relevant features enter the regression model. For the separate feature groups, we used elastic net logistic regression (Friedman et al., 2010, Zou and Hastie, 2005), that uses a combination of an L1 (LASSO; Tibshirani, 1996) and L2 (Ridge; Hoerl and Kennard, 1970) penalty. The L1 penalty tends towards sparse models, including only few features. The L2 penalty tends to include all features, but limits the size of their contributions. Two hyperparameters need to be tuned: the α parameter determines the relative weight of the two different penalties, and λ determines the size of those penalties. For the combined models we used group lasso logistic regression (Simon et al., 2013), which uses an L1 penalty on feature groups and an L2 penalty within the feature groups. The group lasso thereby improves interpretation of the AD classification model, because the L1 penalty on feature groups either entirely includes or excludes feature groups. For the group lasso we only need to tune λ: the size of the penalties.

#### 2.9.2 Cross validation within the training data

To determine the performance of the AD classification models within the training data, we used nested cross validation (Krstajic et al., 2014). Nested cross validation takes into account two potential sources of overfitting. One could either include too many predictors, or overestimate accuracy by looping over all the values of the hyperparameters and only pick the best result. To ascertain that one is not subject to any of these two sources of overfitting, nested cross validation uses an inner loop to tune the hyperparameters and an outer loop to train and test the AD classification model. For both the inner and outer loop we used 10-fold cross validation. We repeated this procedure 10 times to reduce the variance resulting from the random partitioning of the subjects into folds.

#### 2.9.3 Application to memory clinic data

To determine the performance of the AD classification models on the memory clinic data, we fitted AD classification models on the entire training data using optimal hyperparameter settings. These optimal hyperparameters were determined using a single tenfold cross validation. The resulting regression models were directly applied to the MRI features of the memory clinic subjects. This yielded AD scores for the memory clinic subjects.

#### 2.9.4 Model evaluation

To evaluate the results, we made receiver operating characteristic (ROC) curves and calculated the area under the curve (AUC) as a measure of classification performance. The AUC is invariant to the class distribution (Bradley, 1997), which is an advantage, because within the training data the number of control subjects is larger than the number of AD patients. Within the training data we compared the healthy controls with the AD patients, and within the memory clinic data we pairwise compared the SMC, MCI patients and AD patients. The four different patient comparisons, for the nine feature groups plus four combined models, yielded 52 comparisons in total. To test the AUC values against chance, we used a permutation procedure with 10.000 permutations. We combined all 52 comparisons within the same permutation procedure to correct for multiple comparisons. For each permutation we permuted the subjects’ labels, and calculated the AUC value for all 52 comparisons. We only registered the maximum of those 52 AUC values, resulting in a permutation distribution of maximum AUC values. The 52 observed AUC values were compared with this distribution, yielding family-wise error corrected *p*-values.

In addition, we calculated sensitivity, specificity, positive predictive values and negative predictive values. We used a cut-off score of 0.5, such that Subjects with Alzheimer's scores below 0.5 were classified as the less severe disease category, and subjects with Alzheimer's scores above 0.5 were classified as the more severe disease category. For example, in the comparison of subjective memory complainers (SMC) and mild cognitive impaired (MCI) patients, the former is regarded as the less severe disease category and the latter is regarded as the more severe disease category. To evaluate the classification models in the memory clinic data, 0.5 is not necessarily the optimal cut-off score. For example, the subjective memory complainers (SMC) and mild cognitive impaired (MCI) patients are not expected to receive Alzheimer's scores close to either 0 or 1. Consequently, a cut-off score of 0.5 sometimes yields high sensitivity values and low specificity values, or the other way around. In these cases, other cut-off scores might result in a better balance between sensitivity and specificity. We have nevertheless used a fixed cut-off score of 0.5, because it eases the interpretation.

## 3 Results

### 3.1 Correction for scan site

We applied scan site correction to the four memory clinic centers (Fig. S1). Before correction, there are large site effects for the diffusion MRI features, moderate site effects for the anatomical MRI features, and no visible site effects for the rs-fMRI features. These site effects have been removed using the ComBat procedure, leaving no visible site effects between the four memory clinic centers afterwards. We did not correct for scan site differences between the training data and the memory clinic data, because the training data consists of different clinical labels (healthy controls and probable AD) than the memory clinic data (SMC, MCI and possible/probable AD). It is therefore not possible to decide whether differences between these data sets are due to scan site differences, or to differences in clinical groups. The differences between the training data and the corrected test data are largest for the diffusion MRI measures.

### 3.2 Classification results

**Fig. 1:**
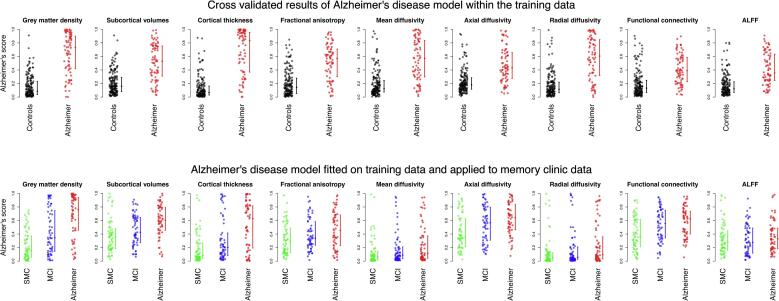
Alzheimer’s disease scores for the feature groups. The top row shows the results on the training data, and the bottom row shows the results on the memory clinic data. The error bars represent the median AD score and the interquartile range. SMC = subjective memory complainers, MCI = mild cognitive impairment, ALFF = amplitude of low frequency fluctuations.

**Fig. 2:**
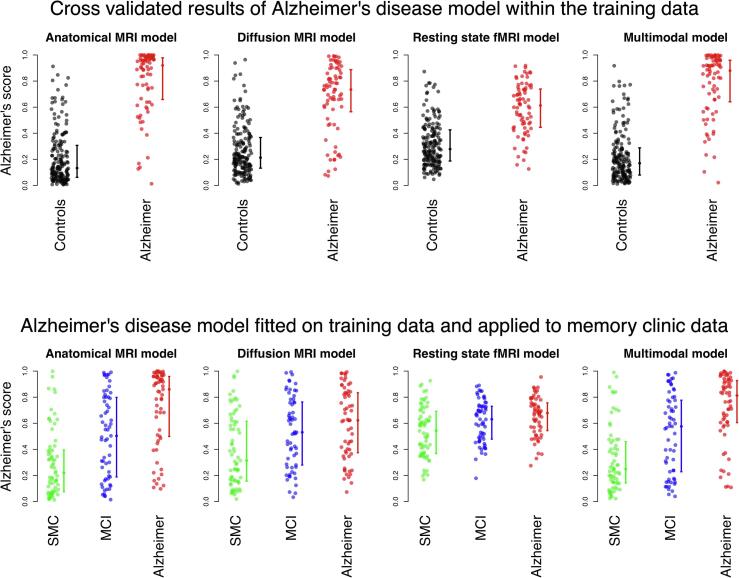
Alzheimer’s disease scores for the combined models. The top row shows the results on the training data, and the bottom row shows the results on the memory clinic data. The error bars represent the median AD score and the interquartile range. SMC = subjective memory complainers, MCI = mild cognitive impairment, ALFF = amplitude of low frequency fluctuations.

The single feature classification models and the multiple feature classification models yielded individual AD scores for all participants (Fig. 1 and Fig. 2 respectively). To evaluate these classification models, we calculated AUC values (Table 4), sensitivity and specificity values (Table 5) and positive predictive values and negative predictive values (Table S2).

**Table 4:** AUC values for the different MRI-based AD classification models.

	Training data	Memory clinic data		
MRI measure	HC vs AD	SMC vs MCI	MCI vs AD	SMC vs AD
Grey matter density	0.91^***^	0.69^**^	0.70^**^	0.86^***^
Subcortical volumes	0.82^***^	0.62	0.66*	0.76^***^
Cortical thickness	0.92^***^	0.64	0.66*	0.76^***^
**Combined anatomical MRI**	0.94^***^	0.69^**^	0.70^**^	0.85^***^
Fractional anisotropy	0.83^***^	0.60	0.57	0.65*
Mean diffusivity	0.84^***^	0.62	0.55	0.66*
Axial diffusivity	0.81^***^	0.63	0.58	0.72^***^
Radial diffusivity	0.85^***^	0.58	0.57	0.64
**Combined diffusion MRI**	0.87^***^	0.65*	**0.57**	0.71^***^
Functional connectivity	0.79^***^	0.66*	0.54	0.71^**^
ALFF	0.81^***^	0.49	0.56	0.55
**Combined rs-fMRI**	0.85^***^	**0.62**	**0.56**	0.68^**^
**Multimodal MRI**	0.94^***^	0.68^**^	0.69^**^	0.84^***^

HC = healthy controls, AD = Alzheimer’s disease, SMC = Subjective memory complainers, MCI = Mild cognitive impairment, ALFF = amplitude of low frequency fluctuations. *p < 0.05, ^**^p < 0.01, ^***^p < 0.001.

**Table 5:** Sensitivity / specificity values for the different MRI-based AD classification models.

	Training data	Memory clinic data		
MRI measure	HC vs AD	SMC vs MCI	MCI vs AD	SMC vs AD
Grey matter density	0.66 / 0.96	0.39 / 0.84	0.69 / 0.61	0.69 / 0.84
Subcortical volumes	0.51 / 0.91	0.46 / 0.78	0.70 / 0.54	0.70 / 0.78
Cortical thickness	0.70 / 0.95	0.25 / 0.91	0.54 / 0.75	0.54 / 0.91
**Combined anatomical MRI**	**0.88 / 0.89**	**0.51 / 0.81**	**0.74 / 0.49**	**0.74 / 0.81**
Fractional anisotropy	0.61 / 0.90	0.28 / 0.75	0.46 / 0.72	0.46 / 0.75
Mean diffusivity	0.57 / 0.92	0.11 / 0.96	0.20 / 0.89	0.20 / 0.96
Axial diffusivity	0.41 / 0.92	0.59 / 0.69	0.69 / 0.41	0.69 / 0.69
Radial diffusivity	0.62 / 0.94	0.11 / 0.90	0.21 / 0.89	0.21 / 0.90
**Combined diffusion MRI**	**0.75 / 0.87**	**0.56 / 0.63**	**0.61 / 0.44**	**0.61 / 0.63**
Functional connectivity	0.34 / 0.94	0.52 / 0.64	0.59 / 0.48	0.59 / 0.64
ALFF	0.39 / 0.96	0.25 / 0.79	0.25 / 0.75	0.25 / 0.79
**Combined rs-fMRI**	**0.67 / 0.86**	**0.69 / 0.45**	**0.82 / 0.31**	**0.82 / 0.45**
**Multimodal MRI**	**0.84 / 0.88**	**0.56 / 0.81**	**0.84 / 0.44**	**0.84 / 0.81**

HC = healthy controls, AD = Alzheimer’s disease, SMC = Subjective memory complainers, MCI = Mild cognitive impairment, ALFF = amplitude of low frequency fluctuations.

#### 3.2.1 Training data classification using single features

The median AD score for the AD patients is higher than those of the healthy controls for all single feature classification models (Fig. 1, top row). The AUC values for discriminating between AD patients and controls range between 0.79 for functional connectivity and 0.92 for cortical thickness. These AUC values are all above chance level, showing that the classification models work well within the training data itself (Table 4, left side).

#### 3.2.2 Memory clinic data classification using single features

All models, except for the ALFF model, assigned the highest median AD score to the AD patients, followed by the MCI patients and later followed by the SMC subjects (Fig. 1, bottom row). The AUC values for the pairwise discrimination between these three groups are depicted in the right side of Table 4. The discrimination between SMC and MCI patients is above chance level for grey matter density and functional connectivity. The discrimination between MCI patients and AD patients is above chance level for grey matter density, subcortical volumes, and cortical thickness. The discrimination between SMC and AD patients is above chance level for grey matter density, subcortical volumes, cortical thickness, FA, MD, DA, and functional connectivity (Table 4, right side).

#### 3.2.3 Training data classification using multiple features

In order to increase classification accuracy, the feature groups were combined into an anatomical MRI, diffusion MRI, rs-fMRI, and multimodal MRI model. For all combined classification models, the median AD score for the AD patients is higher than those of the healthy controls (Fig. 2, top row). The AUC values for discriminating between AD patients and controls are higher for the combined models than those for the single feature models. The multimodal model does however not improve upon the combined anatomical MRI model (Table 4, left side).

#### 3.2.4 Memory clinic data classification using multiple features

The combined classification models were also applied to the memory clinic data. All models assigned the highest median AD score to the AD patients, followed by the MCI patients and later followed by the SMC subjects (Fig. 2, bottom row). In contrast to the training data, the AUC values of the combined models are most often not higher than the AUC value of the best discriminating single feature group. The AUC only increases when combining the diffusion MRI features in order to classify SMC subjects and MCI patients. For all other combined models, the AUC is either the same or lower (Table 4, right side).

### 3.3 Feature group importance

**Fig. 3:**
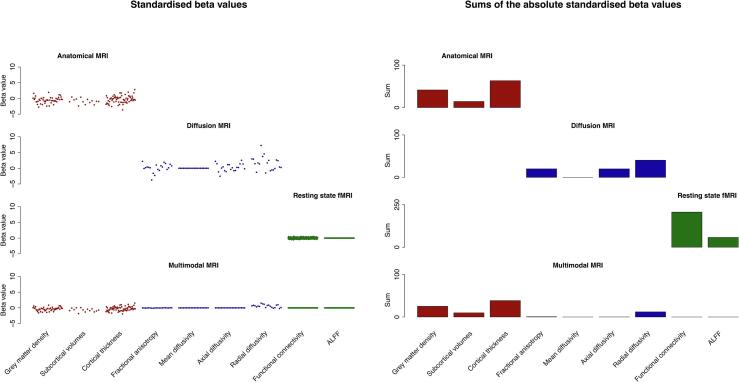
Content of the combined classification models that were fitted on the training data and applied to the memory clinic data. The left panel shows the standardised beta values of the features, and the right panel shows the sums of the absolute standardised beta values per feature group. These plots illustrate the importance of the feature groups for the combined models. The anatomical MRI model takes all three anatomical feature groups into account, the diffusion MRI model takes FA, DA and DR into account, the rs-fMRI model takes both functional connectivity and ALFF into account, and the multimodal MRI model relies mostly on the anatomical MRI features. ALFF = amplitude of low frequency fluctuations.

In order to inspect the contribution of the feature groups to the combined models, we plotted their beta values (Fig. 3). The anatomical MRI model takes all three anatomical feature groups into account, and the largest weight is assigned to cortical thickness. The diffusion MRI model takes FA, DA and DR into account, and disregards MD. The largest weight is assigned to DR. The rs-fMRI model takes both functional connectivity and ALFF into account, but weighs functional connectivity more heavily. The multimodal MRI model relies mostly on the anatomical MRI features, but also includes the DR features.

## 4 Discussion

In this study, we evaluated the generalisability of MRI-based AD classification models. To this end, we used a single center training data set consisting of AD patients and healthy controls, and a multicenter application data set consisting of AD patients, MCI patients and SMC subjects. First, we showed that within the training data there is good classification performance for both the anatomical MRI, diffusion MRI and rs-fMRI models. When a model was trained on one part of the training data, it generalised well to the other part of the training data. Second, we fitted models on the entire training data, and applied those models to the memory clinic data, resulting in AD scores for the memory clinic subjects. As expected, for all three MRI modalities, the AD patients were on average assigned higher AD scores than MCI patients, and the MCI patients were on average assigned higher AD scores than SMC subjects.

There is however large variation in the performance of the different MRI models. The anatomical MRI models generalised best to the memory clinic data. Especially the grey matter density model could differentiate well between all three clinical groups. The cortical thickness model and the subcortical volumes model could differentiate between the AD patients and the other two groups, but not between the SMC subjects and MCI patients.

The diffusion MRI models did not perform as well as the anatomical MRI models. Although classification performance was excellent within the training data for all diffusion MRI measures, there was limited generalisation to the memory clinic data. Possibly, this is due to the fact that white matter alterations in AD mostly occur in the late phase of the disease (Clerx et al, 2012). So, white matter changes might be already present in the probable AD patients from the training data, but these changes might not yet be as large in the MCI patients or possible AD patients from the memory clinic data. Another explanation might lie in the scan site differences for the diffusion MRI measures. It is known that technical variabilities across scan sites can have large effects on diffusion MRI scans (Zhu et al., 2011), and also in the current study the four memory clinic centers largely differed on the diffusion MRI measures. These site differences were removed as much as possible using the ComBat procedure (Fortin et al., 2017, Johnson et al., 2007), but they cannot be removed entirely. Furthermore, we did not remove scan site differences between the training data and the memory clinic data, because the subjects within the training data are not comparable with the memory clinic subjects with regard to their clinical labels. It is therefore not possible to decide whether differences between these data sets should be attributed to scan site differences, or to differences in clinical labels. Yet, it is likely that scan site differences exist between the training data and the memory clinic data, and that possibly they have affected the AD scores of the memory clinic subjects. Diffusion MRI have nevertheless been used successfully in a multicenter AD classification study (Dyrba et al., 2013). However, this study only used probable AD patients and healthy elderly controls, for which differences in white matter are expected to be larger. Furthermore, they used subjects from nine different scan sites, and they achieved the highest accuracy when training and testing was partly done on subjects from the same site. When they trained the model on subjects from eight scan sites, and applied this model on subjects from the ninth scan site, this resulted in lower accuracy.

Regarding the rs-fMRI models, there is a large difference between the functional connectivity model and the ALFF model. The functional connectivity model is somewhat inferior compared to the structural and diffusion MRI measures within the training data, but it generalises reasonably well to the memory clinic data. This model can differentiate between SMC subjects and MCI patients, and between SMC subjects and AD patients. The reasonably good generalisation performance of the functional connectivity model might partly be explained by the absence of large scan site differences. In addition, alterations in functional connectivity likely start in an early phase of AD (Buckner et al., 2005, Sperling, 2011), and this might explain why this model could distinguish reasonably well between SMC subjects and MCI patients. Functional connectivity have previously been shown to be successful for the classification of AD patients, MCI patients and controls in a multi-center setting. However, this was only achieved after employing strict quality measures, including visual inspection of all the data (Teipel et al., 2017). In the current study this was not much of an issue, possibly because we automatically removed noise components with ICA-AROMA (Pruim et al., 2015), and it has been shown that removing ICA based noise components from rs-fMRI data reduces scan site differences substantially (Feis et al, 2015). In contrast to the functional connectivity model, the ALFF model showed very poor generalisation performance. Although the classification performance was good within the training data, this model could not differentiate between any of the three groups within the memory clinic data. This result corresponds to the results of another multicenter study, in which ALFF showed poor classification performance to classify SMC subjects, amnestic MCI patients and AD patients (Teipel et al., 2018).

Combining the MRI features improved the accuracy within the training data, which is a replication of other studies that improved AD classification by combining different MRI measures from the same imaging modality (de Vos et al., 2016, de Vos et al., 2018, Westman et al., 2013), or combining multiple imaging modalities (Dai et al., 2012, Schouten et al., 2016). More importantly, however, this improvement did not translate to the memory clinic data. Some features contributed largely to the combined models, because they had a beneficial effect on AD classification within the training data, but they worsened the results of the combined model on the memory clinic data, because those features did not generalise to the memory clinic data. For example, the combined rs-fMRI model included both functional connectivity and ALFF. Within the training data, this combination increased accuracy compared to both of these features alone. However, within the memory clinic data, this combination decreased accuracy compared to using only functional connectivity. Probably, this is caused by the poor generalisation performance of ALFF.

The classification accuracies within the memory clinic data were substantially lower than those within the training data for all MRI models. These differences can be caused by multiple factors, and we cannot explicitly attribute these differences to any of these different factors. A factor that has likely been important is the difference in clinical populations. It is easier to distinguish AD patients from healthy elderly controls, as in the training data, than to distinguish AD patients from MCI patients and SMC subjects, as in the memory clinic data. In addition, the AD patients in the training data had lower average MMSE scores than the AD patients in the test memory clinic data. The AD patients in the training data were thus clinically more progressed than the AD patients in the memory clinic data. Other factors that might have caused a drop in accuracy from training to test set are scan site differences, differences caused by confounding variables (e.g. age, sex or education) and overfitting on the training data.

We have focused on MRI scans for the AD classification models, although MRI-visible structural and volumetric brain abnormalities occur relatively late in AD (Jack et al., 2010). Amyloid and tau pathology are observable in AD patients well before any pathological change is detectable on a structural MRI scan (Jack et al., 2010). For clinical studies however, structural MRI scans are advantageous, because they are non-invasive and often available. In addition, there is evidence that functional changes as can be seen on a rs-fMRI scan might already occur in an earlier phase of the disease (Buckner et al., 2005, Sperling, 2011). Therefore, rs-fMRI might be sensitive for early detection of AD.

We have only studied AD classification, while memory clinics are confronted with non AD types of dementia as well. In future efforts, to create clinically valuable classification models for more dementia types, it is important to also include non-AD types of dementia.

In conclusion, we studied the generalisation performance of single center MRI-based AD classification models to a multicenter memory clinic data set. The anatomical MRI models generalised best to the memory clinic data, and grey matter density was the best performing anatomical MRI measure. The diffusion MRI models did not generalise well, possibly due to large scan site effects on the diffusion MRI measures, or because white matter alterations mostly occur in progressed AD (Clerx et al, 2012). The functional connectivity model showed reasonable performance for identifying prodromal AD stages, but it was still inferior to the grey matter density model. Moreover, the multimodal MRI model did not improve upon the anatomical MRI model.

## CRediT authorship contribution statement

**Frank Vos:** Conceptualization, Methodology, Formal analysis, Writing - original draft. **Tijn M. Schouten:** Writing - review & editing. **Marisa Koini:** Resources. **Mark J.R.J. Bouts:** Writing - review & editing. **Rogier A. Feis:** Writing - review & editing. **Anita Lechner:** Resources. **Reinhold Schmidt:** Resources. **Mark A. Buchem:** Resources. **Frans R.J. Verhey:** Resources. **Marcel G.M. Olde Rikkert:** Resources. **Philip Scheltens:** Resources. **Mark Rooij:** Writing - review & editing, Supervision. **Jeroen Grond:** Writing - review & editing, Supervision. **Serge A.R.B. Rombouts:** Conceptualization, Writing - review & editing, Supervision, Funding acquisition.

## Declaration of Competing Interest

The authors declare that they have no known competing financial interests or personal relationships that could have appeared to influence the work reported in this paper.

## Figures and Tables

**Supplementary figure 1:**