# Supplementary material for: Pre-trained MRI-based Alzheimer's disease classification models to classify memory clinic patients
Source: Neuroimage Clin. 2020 Jun 4;27:102303. doi: 10.1016/j.nicl.2020.102303 (PMC7303669; doi:10.1016/j.nicl.2020.102303)
Supplement: Supplementary data 1 [file mmc1.pdf]

Supplementary table 1. Sample demographics for the memory clinic centers.

	Memory clinic data			
	Leiden	Maastricht	Nijmegen	Amsterdam
N	40	68	43	38
SMC / MCI / AD	12 / 13 / 15	31 / 24 / 13	16 / 13 / 14	8 / 11 / 19
Sex (♂/ ♀)	20 / 20	42 / 26	25 / 18	30 / 8
Age	70.9 ± 9.0	66.6 ± 11.6	71.6 ± 9.0	65.0 ± 7.5
MMSE	26.4 ± 2.5	27.5 ± 2.6	25.7 ± 2.8	25.4 ± 3.1
CDR	0.59 ± 0.30	0.52 ± 0.17	0.49 ± 0.37	0.63 ± 0.33
GDS	3.9 ± 3.3	3.3 ± 2.6	2.8 ± 1.8	3.5 ± 2.9

Descriptives are presented as frequencies for the categorical variables and as mean ± standard deviation for the other variables. SMC = Subjective memory complainers, MCI = Mild cognitive impairment, AD = Alzheimer's disease, MMSE = mini mental state examination, CDR = clinical dementia rating, GDS = geriatric depression scale.

Supplementary table 2. Positive predictive values / negative predictive values for the different MRI-based AD classification models.

	Training data	Memory clinic data		
	HC vs AD	SMC vs MCI	MCI vs AD	SMC vs AD
Grey matter density	0.81 / 0.88	0.61 / 0.69	0.65 / 0.64	0.76 / 0.79
Subcortical volumes	0.71 / 0.72	0.62 / 0.65	0.62 / 0.61	0.74 / 0.74
Cortical thickness	0.83 / 0.85	0.58 / 0.71	0.65 / 0.69	0.73 / 0.85
**Combined anatomical MRI**	0.88 / 0.78	0.66 / 0.70	0.61 / 0.59	0.77 / 0.78
Fractional anisotropy	0.75 / 0.73	0.51 / 0.50	0.59 / 0.62	0.60 / 0.62
Mean diffusivity	0.74 / 0.76	0.53 / 0.70	0.54 / 0.63	0.58 / 0.80
Axial diffusivity	0.67 / 0.70	0.64 / 0.63	0.55 / 0.54	0.69 / 0.67
Radial diffusivity	0.77 / 0.81	0.51 / 0.50	0.55 / 0.65	0.55 / 0.65
**Combined diffusion MRI**	0.81 / 0.71	0.59 / 0.58	0.52 / 0.52	0.62 / 0.60
Functional connectivity	0.63 / 0.69	0.58 / 0.57	0.53 / 0.53	0.62 / 0.60
ALFF	0.68 / 0.82	0.52 / 0.52	0.50 / 0.50	0.52 / 0.52
**Combined rs-fMRI**	0.76 / 0.67	0.57 / 0.53	0.57 / 0.54	0.63 / 0.57
**Combined multimodal MRI**	0.86 / 0.76	0.68 / 0.72	0.64 / 0.60	0.82 / 0.80

HC = healthy controls, AD = Alzheimer's disease, SMC = Subjective memory complainers, MCI = Mild cognitive impairment, ALFF = amplitude of low frequency fluctuations.
